# Mortality registration and surveillance in China: History, current situation and challenges

**DOI:** 10.1186/1478-7954-3-3

**Published:** 2005-03-16

**Authors:** Gonghuan Yang, Jianping Hu, Ke Quin Rao, Jeimin Ma, Chalapati Rao, Alan D Lopez

**Affiliations:** 1 Chinese Academy of Medical Sciences, Peking Union Medical University, 5, Dong Dan San Tiao, Beijing 100005, China; 2 Center for Health Statistics Information, Ministry of Health, 1, Nanlu, Xizhimenwai, Xicheng District, Beijing 100044, China; 3 School of Population Health, University of Queensland, Public Health Building, Herston Road, Herston, Qld 4006, Australia

## Abstract

**Background:**

Mortality statistics are key inputs for evidence based health policy at national level. Little is known of the empirical basis for mortality statistics in China, which accounts for roughly one-fifth of the world's population. An adequate description of the evolution of mortality registration in China and its current situation is important to evaluate the usability of the statistics derived from it for international epidemiology and health policy.

**Current situation:**

The Chinese vital registration system currently covers 41 urban and 85 rural centres, accounting for roughly 8 % of the national population. Quality of registration is better in urban than in rural areas, and eastern than in western regions, resulting in significant biases in the overall statistics. The Ministry of Health introduced the Disease Surveillance Point System in 1980, to generate cause specific mortality statistics from a nationally representative sample of sites. Currently, the sample consists of 145 urban and rural sites, covering populations from 30,000 – 70,000, and a total of about 1 % of the national population. Causes of death are derived through a mix of medical certification and 'verbal autopsy' procedures, applied according to standard guidelines in all sites. Periodic evaluations for completeness of registration are conducted, with subsequent corrections for under reporting of deaths.

**Conclusion:**

Results from the DSP have been used to inform health policy at national, regional and global levels. There remains a need to critically validate the information on causes of death, and a detailed validation exercise on these aspects is currently underway. In general, such sample based mortality registration systems hold much promise as models for rapidly improving knowledge about levels and causes of mortality in other low-income populations.

## Introduction

Data on the causes, levels and patterns of mortality are critical to support the development of evidence-based health policy. Cause of death statistics represent the longest historical series of data on the health of populations, in some cases extending back well over 150 years [1]. Yet, complete vital registration systems, which have traditionally generated these data, are often difficult and expensive to establish and maintain in developing countries. China is no exception. With 1.3 billion people, complete registration and medical certification of deaths is logistically and financially unattainable at present. However, mortality registration systems have been established in China that provide useful data on the health status of all Chinese, and how it is changing. In this paper, we review the history of vital registration in China, and describe the establishment and operation of the Chinese Disease Surveillance Points (DSP) system. We focus on vital registration because we consider it as the 'gold standard' for mortality statistics, since it provides population level data on causes of death on an annual basis. There are other sources of mortality data such as the census series and annual surveys of population change [2], large scale retrospective household surveys conducted in the periods 1973–75 and 1986–88 [3], and the National Maternal and Child Health Surveillance System [4]. However, censuses and annual population change surveys do not provide routine information on causes of death, retrospective surveys could be affected by recall bias, and the child mortality surveillance system does not inform about causes of death or adult mortality. As a result, owing to these shortcomings, statistics on causes of death from the DSP have been the principal data source for estimating burden of disease in China [5]. Nonetheless, despite the current utility of the DSP system for generating evidence for health policy, there are several challenges yet to be overcome, as discussed in this paper. The primary aim of this paper is to describe the operation, strengths and weaknesses of mortality registration systems in China. Little is known outside of China about the characteristics of such systems and their potential application to other low-income countries.

### History and Function of Vital registration in China

Prior to 1950 vital registration hardly functioned in China, and even then only yielded reports on causes of death for the cities of Beijing and Nanjing[6]. The reported crude death rates ranged from about 18 to 21 per 1000, and the only causes of death reported were tuberculosis, measles, acute infectious disease, 'infant disease', respiratory disease, heart disease, urinary disease, digestive disease, stroke, and ill-defined causes. Cancer was not listed. In 1957, vital registration was expanded to several other large cities, including Shanghai, Tianjin, Harbin, and Wuhan. Thereafter, the vital registration system was extended to include more cities and counties.

In 1976, a nationwide mortality survey was undertaken yielding information on the causes of about 20 million deaths [7]. Data on symptoms experienced by the deceased were collected through retrospective enquiry of all households in China, and based on this information, the cause of death was assigned by a team of physicians. Although this survey was primarily targeted to collect information on cancer mortality, the survey vastly increased the level of appreciation in China about the utility of reliable cause of death data for health planning, and gave impetus to the expansion of vital registration to its current extent. Subsequently, in 1987, the Ministry of Health established a vital registration system to record the fact and cause of death. At present, the vital registration system covers 41 cities (15 large cities and 21 middle/small cities) and 85 counties, among which 25 are 'suburban' counties adjacent to large cities, such as Beijing, Tianjin, and Shanghai. The other counties are located in 12 provinces, mostly in the eastern and central areas of China. Only a few are located in the western regions. The total population covered by this vital registration system in 2000 was about 110 million, half in cities and half living in rural counties. Half of the population covered lives in the eastern region, 40% in the central area, and 10% in the western regions [8].

In brief, the system functions as follows. When a person dies, family members report the death to the registration office of the nearest township hospital in order to get a death certificate, then to the police station to deregister permanent residence and obtain ratification for the burial procedure. Staff in the vital registration office fill in the death certificate based on information from family members, and available medical records or documents. One copy of the death certificate is kept in the registration office, and another is sent to the county Center for Disease Control (CDC), where the cause of death is coded. At the time of inception of the coding system in 1987, a specially designed Chinese Classification of Diseases List consisting of over 500 specific diseases or injuries was used, which could be translated into codes from the Ninth Revision of the ICD [9]. From 1990 onwards, coding was directly based on ICD – 9.

The county CDC then uses a prescribed summary tabulation format to submit monthly reports of deaths by age, sex and cause to the Center of Health Information and Statistics, a Department within the Ministry of Health. This is known as the Ministry of Health – Vital Registration (MOH-VR) system, the annual compilations of which are submitted to the World Health Organization and published by WHO. Since 1996, several cities have started producing local tabulations for internal use.

Quality control measures, including training of staff and development of guidelines and regulations for death registration vary between different areas. Registration in cities is better than in counties, and is better in eastern than western areas. An annual review of data is conducted, and areas that reported implausibly low death rates are excluded from statistical tabulations produced by the MOH-VR system.

### Assessment of Chinese Vital Registration Data

**Figure 1:**
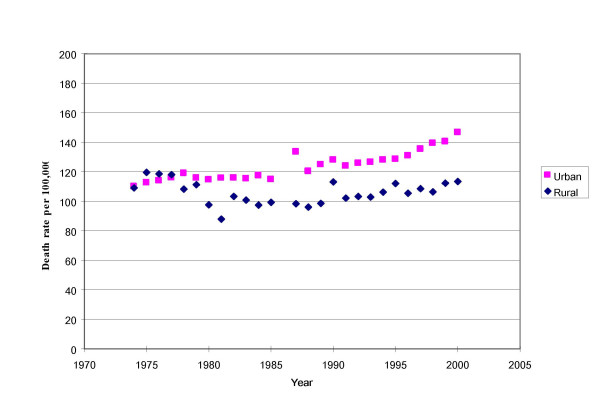
Trends in reported cancer mortality in urban and rural areas of China, 1973–2000

A good test to assess the quality of the vital registration data is to examine trends in cause-specific death rates [10]. For example, the reported death rate from cancer fluctuated improbably in rural areas between 1975 and 1989, then remained relatively stable during the 1990 s (Figure 1). This fluctuation belies an expectation of steady change in cancer mortality over time. Factors causing the fluctuations in cancer mortality trends reported in the vital registration data might include an increase in population coverage with poor quality of data from new reporting areas, or a change in proportions of people accessing health facilities. Another possible reason is that data cleaning was arbitrary, without explicit standards for exclusion of data from specific sites.

An additional limitation of the data system is that birth registration was not included. This undoubtedly contributed to the implausibly low death rates reported for infants in both the MOH-VR data and the DSP system (Table 1), since there was no mechanism for linking infant deaths to births. In comparison, the Census and the National Maternal and Child Health Surveillance system reported much higher infant death rates for the same points in time (see Table 1). Hence, although routine death registration systems provide important information on causes of death, they need to be strengthened to provide a more complete picture on the levels and causes of mortality.

**Table 1:** Unadjusted death rates during infancy (per 1000 population) reported from various data sources in China, 1991 and 2000.

**Data source**	**Total population**		**Urban**		**Rural**	
	
	**1991**	**2000**	**1991**	**2000**	**1991**	**2000**
Census	32.9	32.0	-	-	-	-
NMS	-	-	16.5	8.0	25.4	15.2
DSP	21.4	13.5	8.2	7.7	24.6	14.5
CMSS	50.2	32.2	-	11.9	-	36.4

Source: [4]

**Figure 2:**
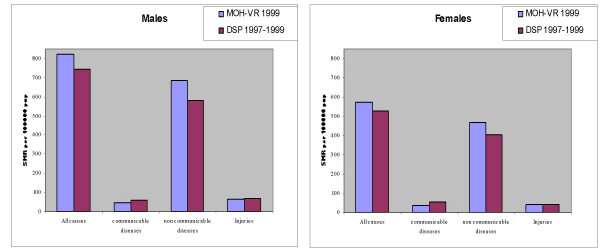
Comparison of age standardized mortality rates* due to broad cause groups, from MOH -VR and DSP systems in China. * Standardized onto WHO World Population [19]

More importantly, the coverage of the MOH-VR system is biased towards the more urban and better-off populations of eastern China. Death rates from infectious disease are lower in the MOH-VR system than those reported by the more representative DSP system, which includes populations in poor rural areas (Figure 2). Similarly, the rates from non-communicable diseases are higher in the MOH-VR system than the DSP system. These observations suggest that the data from the vital registration system are not a true reflection of the mortality profile in China. This concern led to the establishment of the Disease Surveillance Points system as described below

### National Diseases Surveillance Points System (DSP)

In order to improve the usability of data from the vital registration system, the Peking Union Medical University/Chinese Academy of Medical Sciences put forward a proposal in 1978 to develop a sample based Disease Surveillance Point (DSP) system. The system was designed primarily to collect data on births, causes of death, and the incidence of infectious diseases. A pilot study was carried out in East Town and Tongxian counties of Beijing in 1978. The Ministry of Health then instructed departments of health at province and county levels to recognize disease surveillance as an important public health task, and set up disease surveillance points under the technical guidance of the Chinese Academy of Preventive Medicine. By 1989, there were 71 DSPs scattered throughout 29 provinces in the country, with a standard working procedure for data collection, management, analysis and dissemination. However, the system was not representative of the national population [11]. In 1990, the Chinese Academy of Preventive Medicine established a nationally representative population sample of 145 points based on random sampling. This revision of the DSP was an activity under the 'Health I plus' project, supported by a loan from the World Bank.

#### Revised DSP Sampling Plan

Based on the principle that the characteristics of the population under surveillance should be similar to that of the general population in different geographic areas, a multi-stage cluster probability sampling was designed with stratification at three levels.

The first level of stratification was according to 7 geographic regions (Northeast, North, East, South, Southwest, Northwest and Central areas) and 3 municipalities (Beijing, Tianjin and Shanghai) in China. The second level was based on the urban and rural location of primary sampling units. Within rural areas, a third level of stratification was based on a classification of rural sites into four socio economic strata, based on the 1982 Census returns about average levels of variables such as literacy rates, GDP per capita, and dependency ratios. Also, urban areas were re-classified according to population size into big cities, with over 1 million population, middle sized cities with 0.5 -1 million population and small cities with 0.2–0.5 million population.

**Figure 3:**
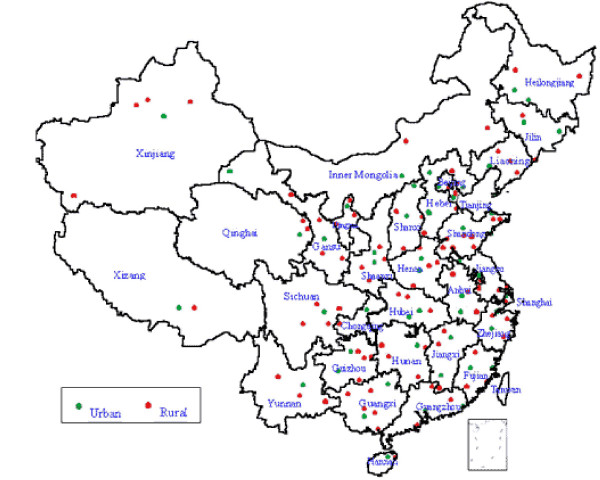
Distribution of sample points in DSP system, China, 2000

The primary cluster unit in urban areas was the city, and in rural areas, the county. Probability proportionate to population size sampling (PPS) was used to select a city or county, using 1982 Census data. In the second stage cluster, in selected cities or counties, the unit of sampling was a 'neighborhood' (Jiedao) within cities, or 'townships' (Xiang) in rural areas. Both the 'Jiedao' and the 'Xiang' represent a community with a primary government, with a population ranging from 30 000 – 100 000. PPS sampling was again used for selection of units at the second stage, such that the probability of selection was according to population size of the neighborhood or township[12] The resultant new DSP system consists of 145 points, which are scattered over the 31 provinces or autonomous regions or municipalities of China (Figure 3).

A population of about 10 million resides in the areas covered by the system (a little under 1% of the Chinese population). Based on national data on public health indicators used for stratification, the selected DSP sites are representative of the national population[12], and the socioeconomic characteristics of these sites derived from the 2000 Census data are shown in Table 2. As expected, a general gradient can be observed in socioeconomic status across the different rural strata, ranging from 1 (best off) to 4 (worst off).

**Table 2:** Socio economic characteristics of sites representing different strata in the DSP (Rural 1 best off; Rural 4 worst off)

**Socioeconomic characteristic**	**Urban**	**Rural 1**	**Rural 2**	**Rural 3**	**Rural 4**
Average GDP* (Million RMB per site)	5098	5108	2602	2054	552
Average literacy rate (%)	91.6	79.5	80.6	78.5	60.5
Average dependency ratio(%)	32.6	44.2	48.4	50.1	57.8
Average Infant mortality rate (per 1000 live births)	9.9	15.8	26.5	42.6	67.8

Source: Chinese Academy of Medical Sciences, 2004, based on data from the 2000 Census

* GDP derived from 1982 Census data on county specific gross agricultural and industrial products. The GDP for each strata was calculated as an average of GDPs for it constituent counties.

#### Mortality registration in the DSP

Since 1990, the system has covered natality, mortality, and the incidence of 35 notifiable diseases. In this section, we describe the process of mortality registration within the DSP system, and comment on aspects regarding quality control of data, particularly with respect to completeness of reporting, and the use of the data for public policy.

In each DSP site, there is at least one township hospital, and the 'Disease Prevention Unit' in these hospitals is responsible for vital registration. The detailed working procedure for mortality registration is described in the guidelines for surveillance in the DSP[9]. In urban areas, almost half of all deaths occur in health facilities, and there are standard protocols for death registration that are closely adhered to. For deaths occurring at home, the attending physician issues a medical certificate of cause of death, in compliance with the registration protocol. Here, we describe briefly the procedure for death registration in rural sites of the DSP.

In rural areas, about 80% of adult deaths occur at home, with few occurring at the township hospital, or other tertiary hospitals in the vicinity. Even for those deaths that occur at home, there is often clinical evidence available from recent consultations with medical staff at township or other hospitals. The procedure for collection and compilation of cause of death data is as follows:

• For deaths occurring at home, a village health worker reports the event to the Prevention Unit at the township hospital. A staff member from the Unit visits the household, and completes a death certificate based on a description of symptoms from family members, and available documents from recent contact with health services.

• For deaths occurring in the township hospital, the DSP staff collect the death certificate from the hospital, completed by the physician who attended the death.

• For deaths occurring in other hospitals, relatives of the deceased submit physician-certified death certificates to the Prevention Unit at the township hospital.

• In the event of a childhood death, or deaths in women of maternal age, the Maternal and Child Health Unit at the township hospital undertakes the investigation of the cause of death, and screens death certificates for such deaths from other hospitals for accuracy.

• Data cleaning and compilation is done at the county or provincial level, and following computerization, an electronic data-file is transferred to the Chinese Academy of Preventive Medicine.

• ICD coding of the underlying cause of death and subsequent tabulation and publication of results is done at the central level in Beijing. Annual reports of deaths by causes, age and sex have been published in Chinese by the Chinese Academy of Medical Science since 1990, and a public access website for these data is currently under development.

There are instances where the above procedures are not strictly adhered.to. In situations where there is a delay in the household investigation by the Prevention Unit staff, family members or neighbours visit the unit to deregister the residential status of the deceased, and obtain the necessary documentation for corpse disposal and other legal purposes. Such instances can promote improper assignment of cause of death in individual cases, since the respondents in these cases may not be familiar with the disease and related conditions experienced by the deceased.

#### Data quality control and improvement

Within the DSP system, there are two methods employed for controlling data quality. The first is an internal procedural check system, which evaluates timeliness of death registration, completeness of entries in the registration form, and the accuracy of data entry. Errors detected from these checks are corrected through re- enquiry, and enhance the usability of the datasets.

At a second level, the datasets are evaluated using statistical measures. The completeness and accuracy of population enumeration in the DSP has been evaluated using the standard United Nations Age Sex Accuracy Index [13], and the results of the evaluation in 1999 are shown in Table 3. The index for almost all regions is around 20, suggestive of accurate age-sex data in the DSP population enumeration (see footnote to Table 3).

**Table 3:** UN Age Sex accuracy Index* for DSP population, by region, 1999

**Region**	**UN Index**
North China	17.7
Northeast China	15.2
East China	21.9
Central China	19.7
South China	22.9
Northwest China	20.5
South West China	23.0

Source: Chinese Academy of Medical Sciences, 2004

The United Nations Index measures the quality of population data as follows: < 20 = accurate; 20 to 40 = inaccurate; > 40 = highly inaccurate [13]

While, there is no mechanism for evaluating the completeness of death registration in the MOH-VR system, the DSP evaluates completeness of both birth and death registration. This is done through independent resurveys, and statistical techniques based on "capture – mark – recapture" methods are used to estimate the completeness of registration [14]. These surveys are conducted once every three years, on a sample of 5000 households in each province.

Results from three such surveys conducted in 1992, 1995 and 1998 are presented in Table 4, for infant deaths and deaths at all ages separately[15,16]. These data suggest that the coverage of infant deaths remains problematic, and as might be expected, is lower than the coverage of adult deaths. Somewhat surprisingly, the extent of undercount was similar in both urban and rural areas, and has shown no improvement in successive surveys. . Although the overall completeness in 1998 was 86 %, there has been no such survey since then, and there is an urgent need to assess coverage in recent years, to ascertain current levels of completeness.

**Table 4:** Estimated under registration of deaths (%) in the DSP system during the 1990 s

**Region**	**1992**		**1995**		**1998**	
	
	Infant deaths	All ages	Infant deaths	All ages	Infant deaths	All ages
**Urban**	-	10.9	25.8	15.1	20.5	13.2
**Rural**	25.4	13.1	35.6	13.0	21.9	14.9
**National**	16.0	12.8	34.7	13.5	20.7	14.1

Source: Calculated from 1992, 1995 and 1998 completeness surveys carried out by the Chinese Academy of Preventive Medicine.

## Discussion

In this paper, we have described in detail for the first time in English, the Disease Surveillance Points System that operates in China, and provides critical information on the health of one-fifth of the world's population, from a sample of less than 1 % of the Chinese population. This is a remarkable achievement, and perhaps the most cost-effective system of data collection to inform health policies and programs ever devised. Yet, the performance, even the existence of the system is not widely appreciated outside of China, despite its obvious implications for rapidly improving knowledge about causes of death in several other low income populations.

Undoubtedly, complete vital registration of deaths with full medical certification is the most appropriate means to monitor the health of populations, but to establish, and particularly to maintain such a system will be outside the realms of possibility for most developing countries for decades to come. Meanwhile, novel, affordable and sustainable approaches to data collection on mortality that is representative of populations are required. The Chinese DSP system described in this paper has many advantages. Almost all countries conduct censuses at least every ten years, and hence the socio-demographic information on which to select a representative sample of surveillance sites is available. Adequate information can be obtained from relatively small samples (≈1% in China and India), and substantial progress has been made with the development of 'verbal autopsy' instruments and procedures to have sufficient confidence in the utility of cause of death data that they produce, at least for broad causes of death. While this may not be sufficient for specific disease or injury control programmes, field experience in Tanzania suggests that the data are useful for determining the need for priority health programs [17].

The data from the DSP have been used to monitor the emergence of tobacco-caused mortality in China[18], and to assess the global and regional burden of disease [5]. Certainly, from a national perspective, much insight has been gained from these data into the levels and patterns of mortality in China over the past decade or so. However, any system that is not based on complete registration and medical certification is of questionable validity for two reasons. Firstly, any undercount of deaths is likely to bias the overall cause of death patterns, with communicable diseases more likely to be missed in poorer segments of the population. Hence, complete registration or at least an assessment of completeness is absolutely necessary in a system like the DSP. Secondly, 'verbal autopsies' are a blunt instrument and can never be expected to capture the full medical history of the deceased. Data generated by systems such as the DSP in China require periodic validation to calibrate the degree of uncertainty in cause of death statistics and to suggest appropriate adjustment factors for specific causes of death.

The authors are currently undertaking such a validation study based on a sample of 2900 deaths in six cities and 3500 deaths in nineteen rural counties in China. In the urban sites of the study, two arms of the project are being implemented. Firstly, medical records of the sampled deaths are being reviewed to develop a reference 'gold standard' diagnosis of the underlying cause of death, using the international form of medical certificate of cause of death. For the same deaths, the diagnosis from the routine registration system is compared and validated against this reference diagnosis, to assess the validity of the routine system. Secondly, for each of these deaths, a verbal autopsy interview was conducted to derive a cause of death, and this will be compared with the reference diagnosis to establish the validity and operational characteristics of the verbal autopsy procedure to be used in the DSP system.

In rural areas, the same standard procedures for verbal autopsy are being introduced, and diagnoses from these standard procedures will be compared with the diagnoses from the routine registration system to measure the reliability of cause of death ascertainment in rural China.

It is envisaged that the results from these studies, as well a proposed under-reporting survey in 2005, will enable correction of datasets from mortality registration systems in China to improve knowledge of cause specific mortality at the population level. The research will also provide the evidence base to strengthen mortality registration in China by identifying structural weaknesses and areas for development, which will minimize undercount and misclassification of deaths in the future. In addition, this evaluation research will build capacity that will result in long term improvements in data quality from the DSP, given the recent changes in the funding, management and coordination of activities within the system. In particular, opportunities to build on existing networks, such as the family planning services system, will need to be more effectively exploited in future to accelerate the implementation of vital registration nationwide.
